# Multiple focal and macroreentrant left atrial tachycardias originating from a spontaneous scar at the contiguous aorta-left atrium area in a patient with hypertrophic cardiomyopathy: a case report

**DOI:** 10.1186/s12872-016-0448-3

**Published:** 2017-01-17

**Authors:** Kyoichiro Yazaki, Yoichi Ajiro, Fumiaki Mori, Masahiro Watanabe, Kei Tsukamoto, Takashi Saito, Keiko Mizobuchi, Kazunori Iwade

**Affiliations:** Department of Cardiology, National Hospital Organization Yokohama Medical Center, 3-60-2 Harajuku, Totsuka-ku, Yokohama-shi, Kanagawa 245-8575 Japan

**Keywords:** Case report, Atrial tachycardia, Contiguous aorta-left atrium area, Spontaneous scar, Hypertrophic cardiomyopathy, 3-D electroanatomical mapping

## Abstract

**Background:**

Spontaneous scar-related left atrial tachycardia (AT) is a rare arrhythmia. We describe a patient with hypertrophic cardiomyopathy (HCM) who developed multiple, both focal and macroreentrant left ATs associated with a spontaneous scar located at the aorta-left atrium (LA) contiguous area.

**Case presentation:**

A 65-year-old man with HCM complained of palpitations. Twelve-lead electrocardiogram showed narrow QRS tachycardia with 2:1 atrioventricular conduction. Two sessions of radiofrequency ablation (RFA) were required to eliminate all left ATs. In the first session, 3-dimensional electroanatomical mapping fused with the image constructed by multi-detector computed tomography showed a clockwise macroreentrant AT (AT1) associated with a low-voltage or dense scar area located along the aorta-LA contiguous area. AT1 was eliminated by RFA to the narrow isthmus with slow conduction velocity within the scar. Additional ATs (AT2-AT4) occurred 1 month after the first ablation. In the second session, AT2 and AT3 were identified as focal ATs with centrifugal propagation and few accompanying fragmentations, and AT4 as a macroreentrant AT with features similar to AT1. AT2 and AT3 were successfully eliminated by performing RFA to the earliest activation site, and AT4 was terminated by performing RFA to the narrow isthmus with slow conduction velocity. No ATs have recurred for 11 months after these RFAs. Interestingly, the substrate for all left ATs was associated with the aorta-LA contiguous area.

**Conclusion:**

To our knowledge, this is the first case of multiple, both focal and macroreentrant left ATs associated with a contiguous aorta-LA spontaneous scar area in a patient with HCM.

**Electronic supplementary material:**

The online version of this article (doi:10.1186/s12872-016-0448-3) contains supplementary material, which is available to authorized users.

## Background

The treatment of atrial tachycardia (AT) is important because, similar to atrial fibrillation [1], AT can lead to poor outcomes in patients with hypertrophic cardiomyopathy (HCM) [2]. While most ATs originating from the left atrium (LA) occur in association with a procedure-related scar due to cardiac surgery or catheter ablation [3–5], left ATs related to a non-procedure-related spontaneous scar have been reported in association with a substrate in the LA anterior wall [6]. A rigid aorta-LA connection exists, which may promote myocardial fibrosis [7, 8]. ATs are often classified according to their endocardial activation pattern as follows: (1) focal ATs, spreading centrifugally from the tachycardia origin based on microreentry, automaticity, or triggered activity, and (2) macroreentrant ATs with a continuous loop of the electrical wavelet based on macroreentry [9]. Scar-related ATs can be classified as either pattern. However, clinical reports particularly concerning spontaneous LA scars are limited.

Here, we describe a patient with HCM who had both focal and macroreentrant multiple ATs associated with a spontaneous scar at the contiguous aorta-LA region that were successfully ablated using 3-D electroanatomical mapping fused with the image constructed using multi-detector computed tomography (MDCT).

## Case presentation

A 65-year-old patient with HCM and a history of common atrial flutter ablation was referred to our clinic with complaints of recurrent shortness of breath and palpitations. Two years prior, echocardiogram showed HCM with a preserved left ventricular ejection fraction and a slightly enlarged LA; coronary angiogram showed intact coronary arteries, and results of a right ventricular myocardial biopsy showed mildly hypertrophic myocardium with mild fibrosis at the subendomyocardium and peri-vascular area. A concomitant cavotricuspid-isthmus-dependent atrial flutter was eliminated by performing linear ablation between the tricuspid annulus through the inferior vena cava during the same hospitalization.

**Fig. 1:**
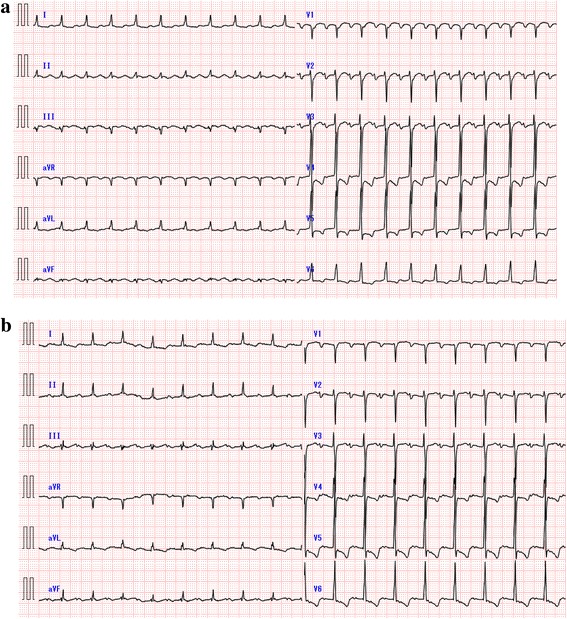
**a** 12-lead electrocardiogram (ECG) showing narrow QRS tachycardia with 2:1 atrioventricular conduction. A saw-tooth wave was detected by the inferior lead, and the tachycardia cycle length (TCL) was 240 ms. **b** ECG showing narrow QRS tachycardia with 2:1 atrioventricular conduction. P-wave deflection was negative in V_1_, and the TCL was 286 ms

**Fig. 2:**
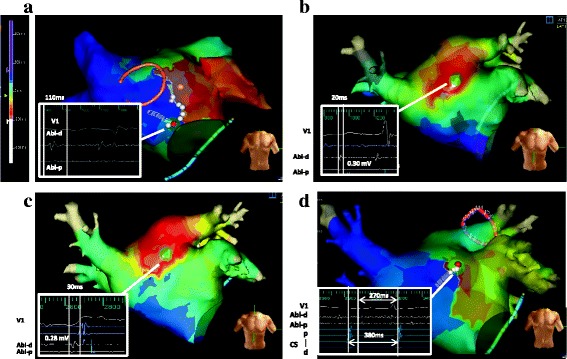
Activation mapping during multiple atrial tachycardias: atrial tachycardia (AT)1 (**a**), AT2 (**b**), AT3 (**c**) and AT4 (**d**). The activation patterns of AT1 and AT4 showed macroreentry. AT1 and AT4 were terminated by performing radiofrequency ablation at the *red circle* where a long-duration, fractionated potential was recorded. The *white circles* represent the unsuccessful site for terminating AT. AT2 and AT3, which had a centrifugal pattern, were terminated at the early activation site where the local potentials preceded the P-wave onset. Abl-d = distal ablation electrode; Abl-p = proximal ablation electrode; CS-p = proximal coronary sinus electrode; CS-d = distal coronary sinus electrode

**Fig. 3:**
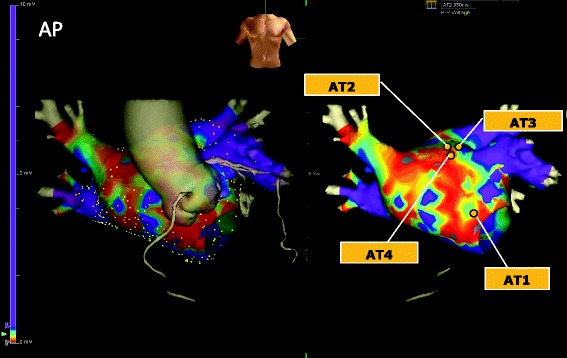
Electroanatomical voltage mapping fused with MDCT. A low-voltage area existed along the aorta-left atrium contiguous region. Termination of the atrial tachycardias (AT1-4) was achieved at the points indicated by the *yellow circles*

Two years later, he again developed atrial arrhythmia. Twelve-lead electrocardiogram showed AT (AT1) with 2:1 atrioventricular conduction (Fig. 1a). An electrophysiological study was subsequently conducted. A 10-polar electrode catheter (Response™, St. Jude Medical Co., Ltd., Minnesota, USA) was placed in the coronary sinus (CS), and a 20-polar electrode catheter (LiveWire™, St. Jude Medical Co., Ltd.) was placed along the tricuspid annulus. Intracardiac electrograms were filtered at 50–500 Hz. Electroanatomical mapping was performed using a 3-D electroanatomical mapping system (Ensite NavX™, St. Jude Medical Co., Ltd.). AT1 persisted from the beginning of the first session with a tachycardia cycle length (TCL) of 248 ms. Because the atrial activation pattern in the CS electrode was detected distal to the proximal sequence and the CS distal activation was earlier than the earliest activation site within the right atrium, we concluded that AT1 originated from the LA. A multipolar ring catheter (Reflection spiral™, St. Jude Medical Co., Ltd.) and a 4-mm open-irrigated-tip catheter (FlexAbility™, St. Jude Medical. Co., Ltd.) were inserted into the LA through a single transseptal puncture. Further activation mapping of the LA demonstrated that AT1 propagated in a figure-eight configuration around the mitral annulus clockwise, and the total activation time of AT1 accounted for almost the entire TCL (Additional file 1; Fig. 2a). 3-D electroanatomical voltage mapping fused with the constructed MDCT image showed a low-voltage (<0.5 mV), dense scar (<0.05 mV) area along the aorta-LA contiguous area from the mid-LA anterior wall through the anterior mitral annulus (Fig. 3); activation mapping indicated that this spontaneous scar area was related to the slow conduction zone for the AT1 circuit. We concluded that AT1 was a macroreentrant AT dependent on the isthmus located along the aorta-LA contiguous area; subsequent radiofrequency ablation (RFA) confirmed the existence of the circuit point-by-point in the entrainment study. AT1 was terminated by RFA at the site near the mitral annulus where a preceding local potential 110 ms before the P-wave onset and post-pacing interval were equal to the TCL during the entrainment study. AT1 and the other ATs could not be provoked again during this session by burst pacing with or without isoproterenol infusion. Due to the severity of the patient’s heart failure, a minimally invasive procedure was preferred; hence, additional RFA was not conducted during the first session.


Additional file 1. Propagation mapping of atrial tachycardia 1. (MPG 2006 kb)

One month later, another AT with a longer TCL of 286 ms (AT2) occurred (Fig. 1b), and the patient developed worsening heart failure. A second electrophysiological session was performed with the same system settings. AT2 was reproducibly provoked by atrial burst pacing without isoproterenol infusion. Activation mapping showed a centrifugal pattern with the origin located at the border of the aorta-LA contiguous, low-voltage area of the mid-anterior LA wall, different from the prior ablation points for AT1 (Additional file 2). Several entrainment studies could not demonstrate manifest entrainment, and there was no fragmented potential that accounted for almost the entire TCL around the earliest activation site. AT2 was terminated by performing RFA at the earliest atrial activation site (Fig. 2b). A third AT with a TCL of 330 ms (AT3) was easily and reproducibly provoked by burst pacing without isoproterenol infusion. AT3 also had centrifugal propagation similar to AT2 (Additional file 3). AT3 was successfully terminated by performing a single RFA at the earliest activation site near the AT2 ablation site (Fig. 2c). A fourth AT with a TCL of 350 ms (AT4) was also provoked by burst pacing without isoproterenol infusion; however, electroanatomical activation mapping of AT4 showed macroreentry in a clockwise fashion, mimicking peri-mitral flutter, similar to AT1 (Additional file 4; Fig. 2d). The critical isthmus of AT4 was located near the upper edge of the prior ablation area for AT1 where the long-duration, fractionated potential that accounted for about 70% of the TCL was recorded and the post-pacing interval was equal to the TCL during the entrainment study. This narrow isthmus was located between the dense scar and upper edge of a prior ablation area. A single RFA at this site terminated AT4, and AT4 was never provoked again by burst pacing with or without isoproterenol infusion. We aimed to achieve noninducibility of ATs, instead of creating a complete block line, because minimal procedures were required due to the severity of the patient’s heart failure.


Additional file 2. Propagation mapping of atrial tachycardia 2. (MPG 874 kb)



Additional file 3. Propagation mapping of atrial tachycardia 3. (MPG 1381 kb)



Additional file 4. Propagation mapping of atrial tachycardia 4. (MPG 1277 kb)


No ATs have recurred in this patient, up to 11 months after these interventions.

## Discussion

The present case demonstrates two important issues: (1) a large spontaneous scar can exist along the aorto-LA contiguous region in the LA that can be arrhythmogenic; (2) multiple ATs with two different activation patterns - both focal and macroreentrant - can occur in association with a spontaneous scar in the LA.

In contrast to the right atrium, the LA has few anatomical obstacles [10]. However, a spontaneous scar can arise in the LA and can act as an arrhythmia substrate [11]. The most common region for a spontaneous scar to develop is the aorta-LA contiguous area; in this region, rigid contact between the aorta and LA exits, which can promote fibrosis and lead to scar formation [12, 13]. Hori Y. et al. demonstrated 68% of the LA very low voltage area (<0.2 mV) overlapped with areas of the LA that contact external anatomical structures, such as the aorta and vertebra, suggesting that contact with external anatomical structures may influence scar formation [13]. Wakabayashi Y. et al. reported a patient with HCM who had a spontaneous scar in the LA anterior wall in contact with the right pulmonary artery, implying that HCM-induced pressure overload might contribute to remodeling and fibrosis [6]. In the present case, heterogeneous myocardial damage, including a wide aorta-LA contiguous scar area and electrically normal posterior LA wall, was observed, suggesting that mechanical stress due to the rigid connection played a more important role than pressure overload in fibrosis and scar formation of the LA. Interestingly, HCM itself plays a potential role in promoting myocardial fibrosis and scar formation. In addition to the genetic background of HCM, it has been reported that transforming growth factor-β1, a potent stimulator of collagen-producing cardiac fibroblasts that also stimulates the differentiation of fibroblasts into more active myofibroblasts, is highly expressed in the myocardium of patients with HCM, implying that HCM increases the susceptibility to triggers that promote myocardial fibrosis [14–17]. Therefore, when cardiologists plan a therapeutic strategy for a patient with HCM who has various left ATs, they should consider the possibility of an existing spontaneous scar and its possible role as an arrhythmogenic substrate even if the patient has no history of surgical interventions to the LA.

Regarding the mechanism of AT from an electrophysiological aspect, macroreentry and microreentry have been reported in association with a spontaneous scar [7, 11]. In cases of spontaneous scar, the aorta-LA contiguous scar area has been reported to be involved in the reentry circuit of localized reentrant AT [12]. However, other mechanisms, such as automaticity and triggered activity, may also be involved in the arrhythmogenesis of ATs due to scar [18]. Furthermore, the mechanical stretch induced by LA enlargement itself may cause changes in cellular action potential and calcium current, which potentially alter the arrhythmogenicity of the tissue [19]. Considering the underlying mechanism of ATs in the present case, AT1 and AT4 (recurrence of AT1) were based on macroreentry, as depicted on activation mapping. AT2 and AT3 were focal ATs depicted as centrifugal propagation. After assessing the underlying mechanism of AT2 and AT3 in the present case, the following observations suggest the likelihood of triggered activity other than microreentry or automaticity: (1) AT2 and AT3 were easily provoked by burst pacing without isoproterenol; (2) no gradual acceleration and/or slowing, known as the warm up or cool down phenomenon, was observed; (3) no manifest entrainment was observed; and (4) no fractionated potential accounting for almost the entire TCL was observed around the earliest activation site of those ATs. While it is often difficult to identify the underlying mechanism of ATs in clinical practice [20, 21], we consider it important to assess the arrhythmia etiology and its mechanisms in order to provide appropriate comprehensive treatment. Because the present case implies the possible arrhythmogenesis of focal ATs, unlike microreentry from a spontaneous scar in the LA, we considered it important to remember that multiple ATs of various mechanisms can arise in association with a spontaneous scar.

The 3-D electroanatomical mapping fused with the image constructed by MDCT is the preferred modality for visualization of tachyarrhythmia propagation in relation to anatomical information. This modality is useful not only in planning the ablation strategy but also in understanding the relationship between anatomical obstacles and the injured myocardium, including low-voltage or silent areas as encountered in the present case. Therefore, we think it is best to use 3-D electroanatomical mapping fused with the image constructed by MDCT when planning ablation treatment for patients with multiple and various ATs associated with an atrial scar.

The case information presented here is beneficial to cardiologists, who should be aware of the possibility that focal and macroreentrant left ATs can occur in association with a spontaneous scar, especially in patients with HCM, even if they have no history of invasive intervention to the LA.

## Conclusion

We have reported a case of multiple LA-AT associated with the aorta–LA contiguous low-voltage area and apparently involving various kinds of pathophysiology. Three-dimensional electroanatomical mapping fused with multidetector computed tomography is useful for visualizing the relation between the aorta and the LA low-voltage area.

## Abbreviations

AT: Atrial tachycardia
CS: Coronary sinus
ECG: Electrocardiogram
HCM: Hypertrophic cardiomyopathy
LA: Left atrium
MDCT: Multi-detector computed tomography
RFA: Radiofrequency application
RFCA: Radiofrequency catheter ablation
TCL: Tachycardia cycle length
